# Carcass Characteristics, Proximate Composition, Fatty Acid Profile, and Oxidative Stability of *Pectoralis major* and *Flexor cruris medialis* Muscle of Broiler Chicken Subjected to with or without Level of Electrical Stunning, Slaughter, and Subsequent Bleeding

**DOI:** 10.3390/ani11061679

**Published:** 2021-06-04

**Authors:** A. B. M. Rubayet Bostami, Hong-Seok Mun, Muhammad Ammar Dilawar, Kwang-Soo Baek, Chul-Ju Yang

**Affiliations:** 1 Animal Nutrition and Feed Science Laboratory, Department of Animal Science and Technology, Sunchon National University, 255 Jungang-ro, Suncheon-si 57922, Jeollanam-do, Korea; rubayet@bsmrau.edu.bd (A.B.M.R.B.); mhs88828@nate.com (H.-S.M.); ammar_dilawar@yahoo.com (M.A.D.); 2 Department of Animal Science and Nutrition, Bangabandhu Sheikh Mujibur Rahman Agricultural University, Gazipur 1706, Bangladesh; 3 Interdisciplinary Program in IT-Bio Convergence System (BK 21 Plus), Sunchon National University, 255 Jungang-ro, Suncheon-si 57922, Jeollanam-do, Korea; gwangsu1649@daum.net

**Keywords:** electrical stunning, neck cutting, bleeding efficiency, meat composition, fatty acid profile, meat oxidative stability, broiler chickens

## Abstract

**Simple Summary:**

The rapid growth in the global and Muslim population has increased the demand for ritually obtained meat, creating a scope of the global meat business. Pre- and post-slaughter practices are of concern in the global meat industries. Therefore, this study examined four types of slaughter, applied with or without a level of electrical stunning, halal neck cutting, and subsequent bleeding (LSHS, MSHS, and HSHS vs. NSHS). Treatments were as follows: (1) NSHS (without electrical stunning, halal neck cut, and subsequent bleeding was for 180 s), (2) LSHS (electrically stunned at 250 mA for 5 s, halal neck cut, and subsequent bleeding was for 180 s), (3) MSHS (electrically stunned at 500 mA for 10 s, halal neck cut, and subsequent bleeding was for 180 s), and (4) HSHS (electrically stunned at 1000 mA for 20 s, halal neck cut, and subsequent bleeding was for 180 s). Slaughtering practices were evaluated in broiler chickens to determine if they influence the carcass characteristics, livability, bleeding out, *Pectoralis major* and *Flexor* *cruris medialis* proximate composition, cholesterol content, fatty acid profile, post-mortem pH, microbial loads, and oxidative stability. With or without stunning, halal neck cutting and subsequent bleeding did not have a significant negative impact on the nutritional aspects, such as proximate composition, cholesterol content, and fatty acid profile, or post-mortem pH, microbial loads except for a variation in some carcass characteristics, livability, bleeding out, and oxidative stability during post-mortem. Since the pre-slaughter conscious state of the animal/bird and post-slaughter bleeding are ritual demands in this process, higher livability and higher bleed out was exhibited in NSHS and LSHS, and there was no large negative impact on nutritional aspects. Therefore, the meat industry can consider without stunning (NSHS) or short-time electrical stunning (LSHS) to capture the global meat market.

**Abstract:**

With an emphasis on the global meat market and considering the ritual requirements and quality aspects, four types of slaughtering treatments were compared: (1) NSHS (without electrical stunning, halal neck cut, and subsequent bleeding for 180 s), (2) LSHS (electrically stunned at 250 mA for five seconds, halal neck cut, and subsequent bleeding for 180 s), (3) MSHS (electrically stunned at 500 mA for 10 s, halal neck cut, and subsequent bleeding for 180 s), and (4) HSHS (electrically stunned at 1000 mA for 20 s, halal neck cut, and subsequent bleeding for 180 s). Four hundred 36 day-old Ross 308 broiler chickens (body weights of 1.4 to 1.8 kg) were divided into four random groups of 100 birds each (ten replicated pens of ten birds). This study examined the livability, bleeding out, *Pectoralis major* and *Flexor cruris medialis* proximate composition, cholesterol content, fatty acid profile and post-mortem pH, microbial loads, and oxidative stability. The livability and bleeding out were higher in NSHS and LSHS than MSHS and HSHS (*p* < 0.05). The *Pectoralis major* and *Flexor cruris medialis* proximate composition, cholesterol content and fatty acid profile, post-mortem pH, and microbial loads were unaffected by the slaughter treatments (*p* > 0.05), but the oxidative stability of *Pectoralis major* differed during the eight-day post-mortem period (*p* < 0.05). The results suggest that for capturing the global meat market, the meat industry can consider NSHS and LSHS because the ritual requirements are fulfilled, and there is no negative impact on the nutritional aspects.

## 1. Introduction

Recently, the quality and safety of food from either animal or plant origins have become increasingly important. This has resulted from the elevated consciousness and appeal of consumers who envisage food characterized by momentous dietetic or health properties [1]. The quality and safety of animal origin food, such as meat or milk, is affected by many components, but the most decisive are the type, species, breed, age, sex, feeding regime, growth rate, and animal handling associated with the marketing and slaughtering of animals [2,3,4,5,6]. Meat is usually obtained from the healthy dressed carcass of different livestock and poultry species. The consumer’s imposition regarding meat is that it must be safe and healthy for human consumption, free from spoilage during or after preservation, and show no deterioration of quality. Among the several types of meat, poultry meat is widely accepted and consumed worldwide because of the lower health risks and the absence of significant barriers based on religion or taboos. The consumption of broiler meat is increasing rapidly, and the broiler industry has become a promising enterprise. The rapidly increasing global and Muslim population has led to further demand for broiler meat because of the religious barriers or taboos regarding pork.

On the other hand, concerns regarding the rearing and production of broiler chicken meat, such as animal welfare and religious issues, before or after slaughter are essential to the global market. During the production of broiler chicken meat, there are many steps, including transportation, handling, pre- and post-slaughter care, and management. The handling and stress during pre-slaughter were reported to affect the welfare and meat quality of broilers [7]. Different types of meat from different livestock and poultry are processed using different slaughtering practices. Globalization creates the opportunity to produce meat by applying different stunning and slaughtering practices. The most common ritually prepared meats in the world are halal (lawful, practiced by Muslims) and kosher (ritually pure, practiced by Jews). Slaughtering practices involve different steps, including the conscious and unconscious state of animals, method of neck cutting, and different types of stunning and pithing, some of which include stunning to induce a state of unconsciousness, insensibility, and immobility before slaughter.

Stunning renders the animal unconscious before slaughter and can be accomplished using mechanical, chemical, or gaseous methods. For large animals, captive bolt stunning is usually applied, while electrical stunning is commonly practiced for poultry. Electrical stunning includes head-only electrical stunning, water bath electrical stunning, head-to-cloaca electrical stunning, and head-to-body electrical stunning. The effects of electrical and gaseous stunning have been reported previously in detail [8,9,10,11]. A comparative study of electrical and gaseous stunning suggested no consistent differences in meat color or cooking quality among turkeys subjected to different stunning methods [12]. In commercial poultry processing plants, electrical water bath stunning is commonly applied. In this method, an electrical current is passed through the body and causes direct muscle stimulation [13,14,15].

Differences in the rate of post-mortem glycolysis induced by various stunning conditions affected the meat quality traits, such as texture, color, and tenderness [16,17]. Slaughtering of animals for human consumption is a delicate operation where strict regulations are needed to ensure food hygiene and safety, animal welfare, and the working environment. Several factors associated with the slaughtering process govern the wholesomeness and meat quality. Accordingly, care should be taken when selecting animal production systems as well as pre-and post- slaughter operations [18].

Different defects, such as hemorrhage in the cut-up poultry carcasses caused by stunning and slaughter, have increased in recent observations. The appearance of imperfections can reduce the acceptance by the consumers and cause economic losses [19,20,21]. On the other hand, the stunning of animals or birds has led to controversy among different religious and animal welfare communities that greatly influence the global meat market. The effects of different stunning methods, such as electrical, mechanical, or gaseous stunning and neck cutting of bled and un-bled procedures, on the meat quality parameters were tested in earlier studies in different species of animals.

Stunning has created controversy in religious circles as well with regard to meat quality worldwide. In contrast, general consumers and some communities are not concerned with the halal way of neck cutting with or without stunning. Previous experiments tested existing and modified slaughtering methods in Korean Hanwoo Cattle (KHC) [22]. *Loin eye* muscle color coordination, physicochemical attributes, and sensory evaluation were similar regardless of the slaughter types of halal neck cutting after captive bolt stunning without pithing vs. non-halal neck cutting following captive bolt stunning with pithing. The halal group relative to the non-halal group had no negative impact on the meat yield and qualitative traits but led to a higher loin eye muscle crude ash content. Therefore, they suggested that the halal way of neck cutting following stunning without pithing could be adopted in Korean slaughterhouses with a modification of existing practices [22].

The rapid increases in the global and Muslim population have increased the demand for ritually obtained meat, creating a scope of the global meat business. Pre- and post-slaughter practices are of concern in the case of global meat industries. To capture the global halal meat market, the prerequisite of halal meat is that the animal must be in a conscious state before slaughter and must be allowed proper bleeding post-slaughter. On the other hand, there is a lack of detailed information on the meat composition and quality under fresh or storage conditions following halal neck cutting with or without a level of electrical stunning in different livestock and poultry species. Few studies have examined the halal way neck cutting following no stunning and the different levels of electrical stunning in chickens. Therefore, the present study compared the effects of with or without electrical stunning, halal neck cutting, and subsequent bleeding of broiler chickens on the livability, bleeding out, carcass observations, *Pectoralis major* and *Flexor cruris medialis* proximate composition, cholesterol content, fatty acid profile, post-mortem pH, microbial loads, and oxidative stability.

## 2. Materials and Methods

### 2.1. Broiler Chicken Husbandry Practice

After receiving the chicks from a local hatchery in Daejeon, the health status was checked by a specialist veterinarian, and they were sorted and raised according to the technical support of the Sunchon National University experimental broiler chicken farm, Suncheon, Korea. A corn–soybean meal-based basal diet was formulated to meet the Nutrient Requirements of Poultry (following both the National Research Council, 1994, Washington, DC, USA and Korean feeding standard for Poultry, 2012). Birds were reared in a closed, ventilated, wire-floor caged broiler chicken house (100 cm long × 90 cm wide × 40 cm high) with a floor space of 1125 cm^2^/bird. A total of four hundred Ross 308 broiler chickens were reared for a period of 5 weeks, divided into starter and finisher phase. All birds were reared provided with similar diet to ensure proper nutrition. However, for proper management, birds were randomly allocated into four groups with ten replicated pens of ten birds.

The internal temperature of the broiler chicken house was set and maintained at 34 ± 1 °C for the first week. Subsequently, it was reduced gradually to 23 °C at 3 °C per week, where it was maintained until week 5. The internal relative humidity was maintained at 50 to 60% throughout the experimental period. The cages had a linear feeder in the front and a nipple drinker in the back to provide ad libitum feed intake and free access to water. The health, management, and environmental conditions of the birds were monitored regularly by veterinarians and animal protectionists. All guidelines for the care and use of animals in research set by the Korean Ministry for Food, Agriculture, Forestry and Fisheries (2008) were followed. The chicks were inspected daily, and dead birds were removed after recording the mortality (pen, date, and body weight).

### 2.2. Stunning, Slaughter, Livability, and Bleeding Out in the Case of Broiler Chicken

At the end of week 5, the final body weights of the birds were checked. The birds were selected randomly based on a similar body weight range. A 12 h off feed period with ad libitum provision of water was practiced before stunning and slaughtering. All birds were inspected by animal specialists to ensure there were no deformities or diseased conditions. After rearing for five weeks, the birds were stunned electrically, the neck was cut in a halal way, and bleeding was performed for 180 s. The electrical stunner used in this study was a Poultryman (Whitehead Engineering Ltd., 60 Hayydon Industrial Est, Radstock, Bath, BA3 3RD, UK). The Poultryman stunner is not the same as an electrical water bath stunner commonly used for commercial poultry production, because the stunning of the birds was applied without any water. After rearing for five weeks, 36-day-old birds with a similar weight range were selected (bodyweight between 1.4 and 1.8 kg) and slaughtered according to the four treatments (100 birds in each treatment). The birds were slaughtered using the following methods: (1) NSHS (without electrical stunning, halal neck cut, and subsequent bleeding for 180 s), (2) LSHS (electrically stunned at 250 mA for five seconds, halal neck cut, and subsequent bleeding for 180 s), (3) MSHS (electrically stunned at 500 mA for 10 s, halal neck cut, and subsequent bleeding for 180 s), and (4) HSHS (electrically stunned at 1000 mA for 20 s, halal neck cut, and subsequent bleeding for 180 s). With or without stunning as well as different time and voltage combinations were applied in the case of stunning of birds to investigate the carcass traits and meat quality attributes in this experiment. Birds were handled gently, and the bodyweight of each bird was recorded before and after slaughter.

After electrical stunning, the birds were observed for 10 to 15 s, and their condition was recorded. The birds were then slaughtered according to the halal method. The livability of the birds before halal neck cutting was recorded. Cutting of the neck was performed with a sharp knife to ensure the jugular veins, trachea, and esophagus were cut. After slaughter, the birds were left hanging for 180 s to ensure proper bleeding. The blood color and amount bled were observed and recorded. Every bird was weighed before and after slaughter. The difference in weight before and after slaughter was considered to be the blood loss. After slaughter, evisceration, skinning, and separation of head and legs the carcass was divided and was kept at 4 °C for cooling for a period of 4 to 6 h. Following cooling, the carcass parts were divided and samples were taken for different analysis. Birds were stunned and slaughtered in the Meat Science Laboratory of Sunchon National University, Korea. The halal slaughtering method applied in this study was according to the humane slaughtering procedure outlined in the Malaysian Standard; MS1500:2009 [23]. Bleeding out was measured based on the amount of blood loss after slaughter, calculated as loss of weight prior to and after slaughter.

### 2.3. Sample Preparation and Carcass Characteristics Observation

After electrical stunning, halal neck cutting, and bleeding, the heads, skins, and internal organs were separated. *Pectoralis major* and *Flexor cruris medialis* samples of chickens from each treatment were collected and stored separately to determine the proximate composition, cholesterol content, fatty acid profile, pH, microbial loads, and oxidative stability (TBARS value). The samples were stored in vacuum packaging at 4 °C temperature. In breast and thigh muscles, the hemorrhages were appraised by a visual grading system, where the breast muscles of the dorsal side of *P. major* and *minor*, and the thigh left and right muscles (medial side) were examined. An independent observation for classification was performed by an expert observer with a keen knowledge of the carcass and meat quality. A threshold model consisting of a discontinuous five-point scale with four cutoff points was used for proper classification. The observations and record of breast and thigh muscles were used to form the cutoff points, showing the particular severity of the hemorrhage: class 1 indicated hemorrhage-free muscles, whereas class 5 indicated muscles with numerous and severe hemorrhages. In the breast part of the carcass, hemorrhage was recorded near the humerus–coracoid joint. The appearance of the carcass and internal organ weight was closely supervised and recorded.

### 2.4. Determination of the Pectoralis Major and Flexor Cruris Medialis Proximate Composition and Cholesterol Content

The chemical compositions of the *Pectoralis major* and *Flexor cruris medialis* samples were analyzed in triplicate for the crude protein (CP), ether extract (EE), moisture, and ash, as described by AOAC [24]. The *Pectoralis major* and *Flexor cruris medialis* cholesterol content was measured using the procedure described by Ahmed et al. [25]. Briefly, the cholesterol was determined from fat, which was segregated via the extraction of 5 g of minced meat (mixed with reference material; 0.5 mL of 5a-cholesterol) with a chloroform and methanol mixture (2:1 vol:vol; as decribed by Folch et al. [26]. The cholesterol was separated from fat using the modified method described by King et al. [27]. The cholesterol was separated from fat after saponification with KOH and extraction with ethyl ether. The samples were then subjected to chromatographic analysis in a DS 6200 gas chromatograph (Donam Co., Seongnam, Gyeonggi-do, Korea) with a flame ionization detector and a Hewlett Packard HP-5 capillary column (J&W Scientific, Folsom, CA, USA) 30 m in length with a 0.32 mm internal diameter and a 0.25 µm polyethylene glycol film thickness. The carrier gas used in this analysis was nitrogen gas. The initial oven temperature was held at 250 °C for two minutes, increased by 15 °C/min to 290 °C (held for 10 min), and then by 10 °C/min to a final temperature of 310 °C (held for 10 min). The other chromatographic conditions were as follows: the split ratio was maintained at 50:1; the injected sample volume was 2 µL; and the injector and detector temperatures were maintained at 280 °C. The cholesterol content of the *Pectoralis major* and *Flexor cruris medialis* samples is expressed as mg/100 g meat.

### 2.5. Determination of Pectoralis Major and Flexor Cruris Medialis Fatty Acid Profile

The fatty acid compositions of *Pectoralis major* and *Flexor cruris medialis* were determined using a direct method for fatty acid methyl ester (FAME) synthesis using a slight modification of the method reported by O’Fallon et al. [28] and Bostami et al. [29]. Briefly, 1 g of minced meat sample was placed into a 15 mL Falcon tube, after which 0.7 mL of 10 N KOH in water and 6.3 mL of methanol were added. The tube was then incubated in a 55 °C water bath for 1.5 h with vigorous hand shaking for 10 s every 30 min to permeate, dissolve, and hydrolyze the sample. After cooling to below room temperature in a cold tap water bath, 0.58 mL of 24 N H_2_SO_4_ in water was added. The tube was then mixed by inversion, after which K_2_SO_4_ precipitated. The sample containing the precipitate was incubated again in a 55 °C water bath for 1.5 h with vigorous hand shaking for 10 s every 30 min. After FAME synthesis, the tube was cooled in a cold water bath. Subsequently, 3 mL of hexane was added, and the tube was vortexed for 5 min on a multitube vortexer. The tube was then centrifuged for 5 min at 3000× *g* (HANIL, Combi-514R, Seoul, Korea), after which the top (hexane) layer containing the FAME was dehydrated through the anhydrous Na_2_SO_4_. The extracted and dehydrated hexane was then concentrated to 1.5 mL and placed into a GC vial for analysis. The methodology followed the detailed procedure described by Bostami et al. [29]. The fatty acid content was expressed as g/100 g of meat.

### 2.6. Pectoralis Major and Flexor Cruris Medialis pH and Microbial Loads

The pH of *Pectoralis major* and *Flexor cruris medialis* was measured using a digital pH meter (Docu-pH+ meter, Sartorius, Goettingen, Germany). First, 3 g of either *Pectoralis major* or *Flexor cruris medialis* meat samples was placed in a falcon tube, after which 27 mL of Deuterium Depleted Water (DDW) was added. The resulting mixture was then homogenized properly. After homogenization and filtration, the pH was measured and recorded. A similar procedure was applied to measure the pH of *Pectoralis major* and *Flexor cruris medialis* meat samples up to 15 days post-mortem. The pH meter with a glass electrode was standardized for pH 4.0 and 7.0 using a buffer solution. The pH meter automatically corrected the pH values, considering the muscle temperature.

After a level of electrical stunning and slaughter of the five-week reared similar-weight birds, the *Pectoralis major* and *Flexor cruris medialis* samples were stored for 15 days post-mortem at 4 °C in a refrigerator. The microbial loads were evaluated from three replicate samples from the *Pectoralis major* and *Flexor cruris medialis*. Overall, 25 g of *Pectoralis major* and *Flexor cruris medialis* meat samples were homogenized with 225 mL of a 0.85% (*W/V*) NaCl solution, after which 10-fold serial dilutions (using 0.85% NaCl solution) were plated on Tryptic soy agar. For each dilution, duplicate plates were incubated at 37 °C for 48 h, and the colonies were counted immediately after incubation. After counting the microbial colonies in duplicate incubated agar plates, microbial counts were expressed as log_10_CFU/g.

### 2.7. Pectoralis Major and Flexor Cruris Medialis Oxidative Stability

The meat oxidative stability of broiler chickens was determined by preserving the *Pectoralis major* and *Flexor cruris medialis* samples in a refrigerator at 4 °C for 15 days post-mortem. The thiobarbituric acid reactive substances (TBARS) were then determined. Briefly, 4 g of *Pectoralis major* and *Flexor cruris medialis* meat samples were homogenized with a homogenizer (Ultra-Turrax T-25 Basic, IKA Werke, GMBH & CO. KG, Staufen, Germany) at full speed for 1.5 min in 10 mL of a solution containing 20% trichloroacetic acid (TCA) in 2 M phosphoric acid and 10 mL distilled water. The mixture was then filtered through Hyundai Micro No. 60 (Hyundai Micro Co. Ltd., Chungmu-ro Jung-gu, Seoul, Korea) filter paper. Equal amounts of filtrate (2 mL) and 2-thiobarbituric acid (98% 4, 6 dihydroxy-2-mercaptopyrimidine, 0.005 M in DW) were heated in a shaking water bath at 80 °C for 30 min. After cooling, the absorbance was measured at 530 nm using a VIS-Spectrophotometer (Libra S22, Biochrom Ltd. Cambridge, UK). The TBARS value was then calculated as follows: TBARS vale = {(Sample absorbance value-Standard absorbance value)/Sample weight} × 3 × 100. The amount of TBARS is expressed as the micromoles of malondialdehyde (MDA) per 100 g of meat.

### 2.8. Statistical Analyses

All data were subjected to ANOVA using the General Linear Models (GLM) function of the Statistical Analysis System (SAS, 2003, Version 9.1, SAS Institute, Cary, NC, USA) [30]. Each cage was considered as the experimental unit for carcass characteristics, proximate analysis, cholesterol content, fatty acid profile, meat pH, microbial loads, and oxidative stability. The means were calculated using the least squares method and presented with the standard error of the mean (SEM). Differences among means were determined by the Student’s t-test. The statistical model used to test the effects of treatment on proximate composition, cholesterol content, fatty acid profiles, meat pH, microbial loads, and TBARS value was:

Yij = μ + αi + eij

where Yij = the response variable, µ = the general mean, αi = the effect of the treatments, and eij = the random error. Student’s t test with a probability level of *p* < 0.05 was used to compare between mean values. A *p* ≤ 0.05 was considered to indicate significance for all analyses, while a *p* < 0.10 was considered as a statistical tendency.

## 3. Results

### 3.1. Carcass Characteristics, Livability, and Bleeding Out

As shown in Table 1, the general carcass characteristics of the broiler chickens were similar regardless of the halal neck cutting with or without a prior level of electrical stunning and subsequent bleeding (NSHS vs. LSHS, MSHS, and HSHS) (*p* > 0.05). On the other hand, some of the internal organ weights of the broiler chickens differed among the slaughter types NSHS vs. LSHS, MSHS, and HSHS (*p* < 0.05). The liver weight was higher in the HSHS group than the NSHS, LSHS, and MSHS groups (*p* < 0.05). The gall bladder weight was higher in the HSHS group than the NSHS and LSHS groups (*p* < 0.05). The large intestine weight was higher in the LSHS and HSHS groups than the MSHS group (*p* < 0.05). The kidney weight was higher in the MSHS and HSHS groups than in the NHS and LSHS groups (*p* < 0.05).

Bleeding out was higher in the NSHS group than MSHS and HSHS (*p* < 0.05) (Table 1). There were no significant differences observed between the NSHS and LSHS groups in bleeding out; however, LSHS and MSHS showed differences from the HSHS group in bleeding out (*p* < 0.05). The appearance of defects was higher in the MSHS and HSHS groups than the NSHS and LSHS groups. In the MSHS and HSHS groups, the blood, liver, and carcass muscle color was blackish red. The carcass breast and thigh meat hemorrhage levels were higher in the HSHS group than the NSHS group (*p* < 0.10). Moreover, livability after different levels of stunning showed that the livability of the NSHS and LSHS birds was higher than the MSHS and HSHS birds (*p* < 0.05).

### 3.2. Pectoralis Major and Flexor Cruris Medialis Proximate Composition, Cholesterol Content, and Fatty Acid Profile

As shown in Table 2, the proximate composition and cholesterol content of the *Pectoralis major* and *Flexor cruris medialis* were similar among the birds subjected to halal neck cutting after level of electrical stunning or no stunning (NSHS vs. LSHS, MSHS, and HSHS) (*p* > 0.05). Table 3 lists the fatty acid profiles. The fatty acid profile of the *Pectoralis major* and *Flexor cruris medialis* was similar regardless of the method of slaughter (NSHS vs. LSHS, MSHS, and HSHS) (*p* > 0.05).

### 3.3. Pectoralis Major and Flexor Cruris Medialis pH, Microbial Loads, and Oxidative Stability

Table 4 shows the pH of the *Pectoralis major* and *Flexor cruris medialis* subjected to halal neck cutting after a level of electrical stunning or no stunning. The pH of the *Pectoralis major* and *Flexor cruris medialis* during post-mortem storage was similar regardless of the method of slaughter (NSHS vs. LSHS, MSHS, and HSHS) (*p* > 0.05).

Table 5 lists the *Pectoralis major* and *Flexor cruris medialis* microbial load. The microbial load in the *Pectoralis major* and *Flexor cruris medialis* during post-mortem storage was similar regardless of the method of slaughter (NSHS vs. LSHS, MSHS, and HSHS) (*p* > 0.05).

As shown in Table 6, there were no significant differences in the TBARS value during one and 15 days post-mortem for *Pectoralis major*. On the other hand, at eight days post-mortem, the TBARS value was lower in the MSHS and HSHS groups than for the LSHS group (*p* < 0.05). The TBARS values of the *Flexor cruris medialis* showed no variations among the groups (NSHS vs. LSHS, MSHS, and HSHS) during one to 15 days post-mortem (*p* > 0.05).

## 4. Discussion

### 4.1. Carcass Characteristics, Livability, and Bleeding Out

Because birds with a similar body weight range were selected and slaughtered after the treatments, there were no differences in the general carcass characteristics. On the other hand, there were some differences in the internal organ weight among the slaughter treatments. The actual reasons for the differences in the internal organ weight are unclear. The electrical stunning frequency with time combination and halal neck cutting might affect the internal organ weight. The differences in the liver, gall bladder, large intestine, and kidney weight among the slaughter groups might be due to the pre-slaughter electrical stunning stress due to the flow of current and time combinations along the bird’s body, and convulsions. Gregory and Wilkins [31] reported a higher frequency of damage to the carcasses when electrical stunning was used. Sances and Larson [32] reported that electrical stunning with an extremely high current might cause problems during the induction of unconsciousness. On the other hand, ventricular fibrillation, cardiac arrest, and physiological activity caused by the frequency of current during electrical stunning might explain the variations in the internal organ weight observed in the current study [33,34]. Inconsistent with the present result, there was no significant impact on the liver in a goose study [35,36].

The total blood loss of the birds was affected by halal neck cutting after electrical stunning and subsequent bleeding (NSHS vs. LSHS, MSHS, and HSHS). The highest and lowest blood loss was observed in the NSHS and HSHS group, respectively. The blood loss from the birds in the NSHS group was different from that in the MSHS and HSHS groups. The LSHS and MSHS groups differed from that of the HSHS group. On the other hand, NSHS and LSHS did not differ significantly. The reason for the difference in blood loss after slaughter with or without electrical stunning might be the livability (higher percentage of birds were alive before slaughter) of the birds and cardiac and circulatory function, which alter the loss of blood from the carcass of broiler chickens. Stunning can result in a loss of blood pressure, heart attack, and loss of oxygen to the brain [37], which would lead to insensibility and aggravation of the birds. These conditions lead to arrest of the cardiac system and less bleeding out after slaughter [38,39]. The association between stunning and bleeding out in broilers, turkeys, and rabbits has been reported earlier [18,39,40], and it was suggested that higher levels of electric stunning affect the avian circulatory system.

The stress and factors that influence the mortality of birds are temperature and RH, daily periods, season, density of broilers per crate, stocking density per lorry, distance between farms and slaughterhouse, pre- and post-slaughter factors, transportation, transport time, lairage, lairage time, and lung congestion [41,42]. Current research has shown that level of electrical stunning with frequency and time, and neck cutting, could affect the welfare, livability, and carcass appearance of birds. Gregory and Wilkins [43] suggested that stunning led to a higher incidence of carcass damage, with approximately 90% of animals undergoing heart fibrillation, which resulted in inefficient bleeding, severe muscle contraction, hemorrhage, and death of the birds, and ultimately lower meat quality. No or lower electrical stunning along with halal neck cutting methods (NSHS and LSHS) might initiate rapid blood flow into the blood vessels before clotting compared to higher stunning and slaughter (HSHS) [44]. The LSHS, MSHS, and HSHS groups showed more than the NSHS group. Raj et al. [17] also reported appearance defects due to different types of stunning.

In addition to the level of electric stunning, multiple factors might be associated with the livability and carcass conditions with hemorrhage. A previous study suggested that electrical stunning can cause the breaking of bones and hemorrhaging of muscles [38], with higher incidences of damage being observed in response to higher levels of electricity [43]. Current research findings also support previous studies, which revealed higher hemorrhage of muscles and carcass damage of the birds subjected to the MSHS and HSHS slaughter group than the NSHS and LSHS groups. Consistently, electrical stunning passes a current through the whole body, which can stimulate the muscles directly, cause convulsions in chickens [45,46], unconsciousness in cattle [47,48] and cause more damage to the carcass [49].

### 4.2. Pectoralis Major and Flexor Cruris Medialis Proximate Composition, Cholesterol Content, and Fatty Acid Profile

Several studies have investigated electrical stunning [11,50], effectiveness of stunning with various concentrations of gases [9,46], the effects of stunning with different types of gases [46,51], and a comparison of gaseous and electrical stunning on post-mortem muscle and meat quality [9,10,17]. On the other hand, halal slaughter following different levels of electrical stunning is another study interest in terms of meat quality for the broiler meat industry based on global halal meat market capture and creation of opportunities. Therefore, this study compared halal neck cutting with or without electrical stunning (NSHS vs. LSHS, MSHS, and HSHS). No variations in the proximate composition of *Pectoralis major* and *Flexor cruris medialis* were observed.

Addeen et al. [44] reported no variations in the protein content in a study of Islamic or halal slaughtering, decapitation, conventional neck cutting, and un-bled slaughter of chickens. Bostami et al. [29] reported no negative impact on meat composition except the ash content in Hanwoo cattle subjected to halal slaughter following stunning with or without pithing. The cholesterol content in the *Pectoralis major* and *Flexor cruris medialis* was unaffected by halal neck cutting after electrical stunning (NSHS vs. LSHS, MSHS, and HSHS). Generally, the genotype or breed, age at slaughter, feeding regime, castration, or other factors can alter the meat or *longissimus thoracic*, *infraspinatus*, and *biceps femoris* muscle cholesterol [52,53].

The current study indicates similar *Pectoralis major* and *Flexor cruris medialis* cholesterol contents regardless of electrical stunning, halal neck cutting, and subsequent bleeding. In cattle, sheep, goats, or poultry, the fatty acid profile of the animal is generally influenced by the body weight, gender and breed of animals, weight of the animal, age at slaughter, geographical factors, genotype, season, and nutritional regime [54,55,56,57]. This study showed that halal neck cutting after electrical stunning (NSHS vs. LSHS, MSHS, and HSHS) did not affect the fatty acid profile of broiler chicken.

### 4.3. Pectoralis Major and Flexor Cruris Medialis pH, Microbial Loads, and Oxidative Stability

The meat pH is related to the biochemical state of the muscle at the time of slaughter and after the development of rigor mortis. This affects both the light reflectance properties of the meat and the chemical reactions of myoglobin [58]. The muscle pH and meat color are highly correlated. A higher muscle pH is associated with darker meat, whereas lower muscle pH values are associated with lighter meat [58,59]. In extremes cases, meat with higher pH values was dark, firm, and dry (DFD), and the lighter meat was pale, soft, and exudative (PSE).

Generally, stunning can suppress the rate of pH decline in the early post-mortem stages, but it does not always affect the ultimate pH [11]. Stunning of broilers can influence adenosine triphosphate (ATP), creatine phosphate (CP), pH, and lactate [60]. Although convulsions affect the post-mortem muscle pH, they might not change the overall pH [17,45]. Hillebrand et al. [45] and Önenç and Kaya [61] reported no variations in the post-mortem pH following different stunning of broilers and cattle. Consistent with previous studies, the present study shows no differences in *Pectoralis major* and *Flexor cruris medialis* pH during one to 15 days post-mortem among the slaughter treatments. Better quality meat pH ranges from 5.3 to 5.8 [62], while the pH of breast and thigh meat of chicken usually ranges from 5.4 to 5.8 for breast meat and from 5.7 to 6.2 for thigh meat [63,64]. The pH values of the present study are within the ranges of standard values reported in earlier research, indicating no negative impact among the levels of electrical stunning and slaughter treatments. Regarding the increasing trend of pH in all groups, there are a number of factors associated with meat pH change during storage such as genetics, pre- and post-slaughter factors, and handling process. However, there were no statistical differences found among the groups and the trend was similar for all groups.

In the current study, higher amounts of blood were retained (due to less blood loss) in the muscles of the birds following different levels of electrical stunning (LSHS, MSHS, and HSHS) compared to the no electrical stunning group (NSHS). The association of the amount of blood remaining in the carcass with the microbial count was reported by Ali et al. [39] in a broiler chicken study and Nakyinsige et al. [18] in a rabbit study. During the investigation of different slaughtering methods (Islamic or halal slaughtering, decapitation, conventional neck cutting, and an un-bled method), a lower total viable microbial count was reported in the halal method [44]. On the other hand, the current study reveals no significant differences in the total microbial loads during post-mortem storage. The results presented in the current study show that samples of broiler chicken meat exceeded the permissible limit (log_10_^7^ cfu/g) of the total bacterial count recommended by the ICMSF [65]. Regarding crossing, the critical levels of microbial loads might be due to cross-contamination during handling, processing, and storage, warranting further detailed study.

Consumers rate the ultimate food quality and safety as the most significant concerns [66]. The spoilage of meat is associated with the characteristics of the meat, lipid oxidation or shelf life, type of microflora, composition of the meat, and environmental conditions under which meat is stored [67,68]. Lipid oxidation and microbial growth are critical limiting factors that determine the safety and oxidative stability of meat. Insausti et al. [69] established a detectable concentration of 5 mg malondialdehyde/kg of meat for humans. Nevertheless, this value is much higher than that indicated to detect oxidized flavors by other authors. Camo et al. [70] reported that in lamb, a TBARS value above 2 was detectable; Greene and Cumuze [71] stated the TBA range of 0.6–2.0 was detectable.

The feeding regime, management factors, pre- and post-slaughter associates, and meat processing can affect the oxidative stability or shelf life of meat. An investigation that compared Islamic slaughtering, decapitation, conventional neck cutting, and an un-bled method showed that TBARS value, as an indicator of oxidative stability or shelf life, was lower in response to Islamic/halal slaughter than the un-bled method [44]. The present study indicates that the level of electrical stunning could affect the oxidative stability of muscle.

The microbial activity is the most important factor responsible for the spoilage of meat, but some other factors are also associated with meat storage. The availability of energy substrates in meat (low molecular weight compounds, such as lactate or glucose) [72,73], as well as the microbial enzyme activity, metabolic byproducts, and proteolytic meat enzymes, can characterize meat spoilage [74]. Moreover, the decreased extract-release volume (ERV) and lactate, as well as elevated pH during storage, can also contribute to the spoilage of meat [75]. Therefore, a combination of several factors might have resulted in variation in the oxidation of meat among slaughter treatments (NSHS vs. LSHS, MSHS, and HSHS) in the present study. Therefore, a more detailed study is warranted to confirm the impact of electrical stunning (with frequency of current and time combination) and neck cutting on the meat oxidative stability or shelf life and the detailed mechanisms by which these changes occur.

## 5. Conclusions

Some carcass characteristics showed differences according to the slaughter types applied to broiler chickens with or without electrical stunning, halal neck cutting, and subsequent bleeding (NSHS vs. LSHS, MSHS, and HSHS). Bleeding out and livability were higher in the NSHS and LSHS group than the MSHS and HSHS group. Hemorrhage and the appearance of carcass defects were higher in the HSHS group than in the NSHS group. The *Pectoralis major* and *Flexor cruris medialis* proximate composition, cholesterol content, fatty acid profile, post-mortem pH, and microbial loads were similar regardless of the level of electrical stunning, halal neck cutting, and subsequent bleeding (NSHS vs. LSHS, MSHS, and HSHS). The *Pectoralis major* and *Flexor cruris medialis* oxidative stability was not affected significantly after one to 15 days post-mortem storage, except for the higher TBARS value being observed in the *Pectoralis major* in the LSHS group compared to the MSHS and HSHS groups during eight days post-mortem storage. Thus, the present research suggests that stunning, halal neck cutting, and subsequent bleeding did not have a significant negative impact on the nutritional composition or post-mortem pH and microbial loads except for a slight variation in oxidative stability. On the other hand, the global halal meat market can be captured by ensuring a higher livability and bleed out (pre-slaughter conscious state of animal/bird and post-slaughter bleeding are ritual demands) as exhibited in the NSHS and LSHS. Therefore, the meat industry can consider without stunning (NSHS) or short-time electrical stunning (LSHS) to capture the global meat market. A future detailed study can confirm the matter of post-mortem oxidative stability.

## Figures and Tables

**Table 1:** Effects of the level of electrical stunning, halal neck cutting, and subsequent bleeding on the carcass characteristics, livability, and bleeding out in broiler chickens.

Item	NSHS	LSHS	MSHS	HSHS	SEM	*p*-Value
Live weight (g/bird)	1517.78	1507.50	1503.33	1510.00	38.01	0.994
Slaughter weight (g/bird)	1460.00	1442.50	1446.67	1453.33	37.83	0.989
Eviscerated weight (g/bird)	1296.73	1295.60	1293.26	1290.07	34.91	0.999
Wings weight (%)	5.94	5.93	5.92	6.1	0.19	0.944
Neck weight (%)	3.62	3.66	3.50	3.68	0.11	0.711
Head weight (%)	2.98	2.97	2.89	2.97	0.07	0.78
Crop (% LW)	0.252	0.297	0.26	0.251	0.02	0.359
Proventriculus (% LW)	0.360	0.388	0.372	0.398	0.015	0.316
Gizzard (% LW)	2.051	2.144	2.09	2.241	0.095	0.543
Heart (% LW)	0.448	0.406	0.454	0.488	0.025	0.165
Liver (% LW)	1.555 ^b^	1.583 ^b^	1.579 ^b^	1.878 ^a^	0.045	<0.0001
Gall bladder (% LW)	0.137 ^b^	0.102 ^b^	0.121 ^ab^	0.141 ^a^	0.009	0.028
Spleen (% LW)	0.064	0.064	0.060	0.069	0.006	0.765
Pancreas (% LW)	0.211	0.190	0.223	0.213	0.013	0.384
Small intestine (% LW)	2.330	2.246	2.261	2.360	0.084	0.747
Large intestine (% LW)	0.136 ^ab^	0.172 ^a^	0.117 ^b^	0.176 ^a^	0.017	0.071
Cecum (% LW)	0.423	0.453	0.45	0.46	0.028	0.802
Kidney (% LW)	0.566 ^b^	0.548 ^b^	0.646 ^a^	0.684 ^a^	0.026	0.002
Abdominal fat (% LW)	1.115	1.171	1.110	1.330	0.112	0.5
Bursa of fabricious (% LW)	0.193	0.168	0.227	0.244	0.025	0.169
Breast meat hemorrhage	2.225 ^b^	2.300 ^b^	2.280 ^b^	2.760 ^a^	0.15	0.060
Thigh meat hemorrhage	1.775 ^b^	2.145 ^ab^	2.205 ^ab^	2.335 ^a^	0.15	0.068
Blood out (ml/bird)	75.00 ^a^	69.00 ^ab^	63.00 ^b^	47.00 ^c^	3.56	<0.0001
Livability	99.80 ^a^	99.00 ^a^	8.60 ^b^	0.50 ^c^	0.34	<0.0001

^a,b,c^ Different superscript letters within the same row indicate the significant differences (*p* < 0.05). SEM = standard error of the mean. Treatments: (1) NSHS (without electrical stunning, halal neck cut, and subsequent bleeding for 180 s), (2) LSHS (electrically stunned at 250 mA for 5 s, halal neck cut, and subsequent bleeding for 180 s), (3) MSHS (electrically stunned at 500 mA for 10 s, halal neck cut, and subsequent bleeding for 180 s), and (4) HSHS (electrically stunned at 1000 mA for 20 s, halal neck cut, and subsequent bleeding for 180 s).

**Table 2:** Effects of the level of electrical stunning, halal neck cutting, and subsequent bleeding on the *Pectoralis major* and *Flexor cruris medialis* proximate composition and cholesterol content in broiler chickens.

Item	NSHS	LSHS	MSHS	HSHS	SEM	*p*-Value
Meat composition (%)						
*Pectoralis major*						
Moisture (%)	75.20	75.42	75.25	75.70	0.28	0.608
Crude protein (%)	26.43	27.38	26.81	27.00	0.54	0.712
Crude fat (%)	0.65	0.75	0.78	0.72	0.09	0.795
Crude ash (%)	1.44	1.45	1.46	1.47	0.04	0.977
Cholesterol (mg/100g)	95.95	108.71	107.25	108.22	12.31	0.607
*Flexor c. medialis*						
Moisture (%)	72.40	72.56	72.36	74.57	0.69	0.152
Crude protein (%)	21.63	21.89	21.47	21.16	0.37	0.626
Crude fat (%)	5.29	6.42	4.72	4.21	0.73	0.213
Crude ash (%)	1.03	1.11	1.10	1.08	0.04	0.585
Cholesterol (mg/100g)	168.07	162.73	159.26	175.95	24.83	0.410

The lack of superscript letters within the same row indicates no significant differences (*p* < 0.05). SEM = standard error of the mean. Treatments: (1) NSHS (without electrical stunning, halal neck cut, and subsequent bleeding for 180 s), (2) LSHS (electrically stunned at 250 mA for 5 s, halal neck cut, and subsequent bleeding for 180 s), (3) MSHS (electrically stunned at 500 mA for 10 s, halal neck cut, and subsequent bleeding for 180 s), and (4) HSHS (electrically stunned at 1000 mA for 20 s, halal neck cut, and subsequent bleeding for 180 s).

**Table 3:** Effects of the level of electrical stunning, halal neck cutting, and subsequent bleeding on the *Pectoralis major* and *Flexor cruris medialis* fatty acid profile (g/100 g of meat) in broiler chickens.

Item	NSHS	LSHS	MSHS	HSHS	SEM	*p*-Value
Fatty acid profile						
*Pectoralis major*						
Total SFA	28.71	29.76	30.58	30.40	0.82	0.381
Total MUFA	33.52	33.21	35.58	36.70	1.67	0.411
Total n-6	31.07	28.79	27.12	28.24	1.57	0.333
Total n-3	3.24	2.86	2.84	2.70	0.20	0.279
Total PUFA	32.02	30.89	28.02	28.31	1.67	0.269
MUFA/SFA	1.17	1.12	1.17	1.21	0.06	0.774
PUFA/SFA	1.12	1.04	0.93	0.93	0.06	0.131
n-6/n-3	9.79	10.89	9.55	10.86	0.79	0.596
*Flexor c. medialis*						
Total SFA	30.54	30.86	31.16	29.99	0.60	0.585
Total MUFA	36.54	36.38	35.35	35.58	1.54	0.933
Total n-6	27.53	28.65	28.65	28.97	1.73	0.935
Total n-3	3.24	3.19	3.05	3.01	0.18	0.779
Total PUFA	30.41	31.68	31.41	31.56	1.93	0.962
MUFA/SFA	1.20	1.18	1.14	1.19	0.06	0.900
PUFA/SFA	1.00	1.03	1.01	1.06	0.07	0.940
n-6/n-3	8.62	9.12	9.56	9.69	0.55	0.498

The lack of superscript letters within the same row indicates no significant differences (*p* < 0.05). SEM = standard error of the mean. Treatments: (1) NSHS (without electrical stunning, halal neck cut, and subsequent bleeding for 180 s), (2) LSHS (electrically stunned at 250 mA for 5 s, halal neck cut, and subsequent bleeding for 180 s), (3) MSHS (electrically stunned at 500 mA for 10 s, halal neck cut, and subsequent bleeding for 180 s), and (4) HSHS (electrically stunned at 1000 mA for 20 s, halal neck cut, and subsequent bleeding for 180 s).

**Table 4:** Effects of level of electrical stunning, halal way neck cutting, and subsequent bleeding on the *Pectoralis major* and *Flexor cruris medialis* pH in broiler chickens.

Item	NSHS	LSHS	MSHS	HSHS	SEM	*p*-Value
Meat pH						
*Pectoralis major*						
1 day	5.57	5.53	5.59	5.54	0.04	0.711
8 days	7.03	6.96	6.90	6.94	0.09	0.789
15 days	7.06	7.03	6.95	6.94	0.08	0.608
*Flexor c. medialis*						
1 day	6.10	6.10	6.09	6.07	0.02	0.851
8 days	6.76	6.78	6.80	6.75	0.09	0.980
15 days	7.13	7.21	7.20	7.25	0.05	0.344

The lack of superscript letters within the same row indicates no significant differences (*p* < 0.05). SEM = standard error of the mean. Treatments: (1) NSHS (without electrical stunning, halal neck cut, and subsequent bleeding for 180 s), (2) LSHS (electrically stunned at 250 mA for 5 s, halal neck cut, and subsequent bleeding for 180 s), (3) MSHS (electrically stunned at 500 mA for 10 s, halal neck cut, and subsequent bleeding for 180 s), and (4) HSHS (electrically stunned at 1000 mA for 20 s, halal neck cut, and subsequent bleeding for 180 s).

**Table 5:** Effects of the level of electrical stunning, halal neck cutting, and subsequent bleeding on the *Pectoralis major* and *Flexor cruris medialis* microbial loads (Log_10_cfu/g) in broiler chickens.

Item	NSHS	LSHS	MSHS	HSHS	SEM	*p*-Value
Meat microbiology						
*Pectoralis major*						
1 day	8.62	8.59	8.70	8.61	0.05	0.509
8 days	9.24	9.21	9.04	9.18	0.09	0.444
15 days	9.17	9.25	9.22	9.31	0.08	0.634
*Flexor c. medialis*						
1 day	8.70	8.67	8.73	8.72	0.03	0.584
8 days	8.96	9.14	9.15	9.10	0.07	0.220
15 days	9.28	9.40	9.34	9.17	0.09	0.418

The lack of superscript letters within the same row indicates no significant differences (*p* < 0.05). SEM = standard error of the mean. Treatments: (1) NSHS (without electrical stunning, halal neck cut, and subsequent bleeding for 180 s), (2) LSHS (electrically stunned at 250 mA for 5 s, halal neck cut, and subsequent bleeding for 180 s), (3) MSHS (electrically stunned at 500 mA for 10 s, halal neck cut, and subsequent bleeding for 180 s), and (4) HSHS (electrically stunned at 1000 mA for 20 s, halal neck cut, and subsequent bleeding for 180 s).

**Table 6:** Effects of level of electrical stunning, halal neck cutting, and subsequent bleeding on the *Pectoralis major* and *Flexor cruris medialis* TBARS value (µmol MDA/100 g of meat) in broiler chickens.

Item	NSHS	LSHS	MSHS	HSHS	SEM	*p*-Value
Meat TBARS						
*Pectoralis major*						
1 day	2.02	2.01	1.92	1.29	0.33	0.381
8 days	4.94 ^ab^	6.15 ^a^	3.21 ^b^	3.64 ^b^	0.75	0.041
15 days	8.82	12.46	10.23	11.44	2.08	0.652
*Flexor c. medialis*						
1 day	2.17	2.47	2.01	2.33	0.41	0.887
8 days	4.64	6.74	5.14	4.04	1.11	0.431
15 days	20.64	25.14	27.41	29.91	3.43	0.295

^a,b^ The different superscript letters within the same row are significantly different (*p* < 0.05). SEM = standard error of the mean. Treatments: (1) NSHS (without electrical stunning, halal neck cut, and subsequent bleeding for 180 s), (2) LSHS (electrically stunned at 250 mA for 5 s, halal neck cut, and subsequent bleeding for 180 s), (3) MSHS (electrically stunned at 500 mA for 10 s, halal neck cut, and subsequent bleeding for 180 s), and (4) HSHS (electrically stunned at 1000 mA for 20 s, halal neck cut, and subsequent bleeding for 180 s).
